# Does the prehospital National Early Warning Score predict the short-term mortality of unselected emergency patients?

**DOI:** 10.1186/s13049-018-0514-1

**Published:** 2018-06-07

**Authors:** Marko Hoikka, Tom Silfvast, Tero I. Ala-Kokko

**Affiliations:** 1 University of Oulu, Medical Research Centre, Research Unit of Surgery, Anaesthesia and Intensive Care and Department of Anaesthesiology, Division of Intensive Care, Oulu University Hospital, PO BOX 21, FI-90029 OYS, Oulu, Finland; 2 University of Helsinki and Department of Anaesthesiology and Intensive Care, Helsinki University Central Hospital, HUS, FI-00029 Helsinki, Finland

**Keywords:** Emergency medical services, Early warning score, Triage

## Abstract

**Objectives:**

The prehospital research field has focused on studying patient survival in cardiac arrest, as well as acute coronary syndrome, stroke, and trauma. There is little known about the overall short-term mortality and its predictability in unselected prehospital patients. This study examines whether a prehospital National Early Warning Score (NEWS) predicts 1-day and 30-day mortalities.

**Methods:**

Data from all emergency medical service (EMS) situations were coupled to the mortality data obtained from the Causes of Death Registry during a six-month period in Northern Finland. NEWS values were calculated from first clinical parameters obtained on the scene and patients were categorized to the low, medium and high-risk groups accordingly. Sensitivities, specificities, positive predictive values (PPVs), negative predictive values (NPVs), and likelihood ratios (PLRs and NLRs) were calculated for 1-day and 30-day mortalities at the cut-off risks.

**Results:**

A total of 12,426 EMS calls were included in the study. The overall 1-day and 30-day mortalities were 1.5 and 4.3%, respectively. The 1-day mortality rate for NEWS values ≤12 was lower than 7% and for values ≥13 higher than 20%. The high-risk NEWS group had sensitivities for 1-day and 30-day mortalities 0.801 (CI 0.74–0.86) and 0.42 (CI 0.38–0.47), respectively.

**Conclusion:**

In prehospital environment, the high risk NEWS category was associated with 1-day mortality well above that of the medium and low risk NEWS categories. This effect was not as noticeable for 30-day mortality. The prehospital NEWS may be useful tool for recognising patients at early risk of death, allowing earlier interventions and responds to these patients.

**Electronic supplementary material:**

The online version of this article (10.1186/s13049-018-0514-1) contains supplementary material, which is available to authorized users.

## Background

Prompt emergency identification and correct risk assessment are corner stones of a successful emergency medical service (EMS) system. Both also affect patient survival. [1] While the use of early warning scores (EWS) has been shown to be feasible for predicting mortality and deterioration of hospitalized patients [2], it is unclear whether the use of early warning scores in the prehospital setting is efficient to detect patients at risk of death. [3, 4] The prehospital research field has mainly focused on the identification and outcome of out-of-hospital cardiac arrest patients as well as other specific patient groups, [5–7] but only few studies have related to the outcome of unselected prehospital patients [8].

In 2012, the use of the National Early Warning Score (NEWS) throughout the entire chain of medical care was recommended by the Royal College of Physicians. [9] Since then, the use of NEWS has been increasingly implemented in hospital wards, emergency departments, as well as in several EMSs in Finland. The use of NEWS in prehospital setting may facilitate earlier identification of patients at risk. In one study, the prehospital NEWS was shown to predict 48-h mortality. [10] However, in daily practice the value of risk assessment to support decision-making in prehospital setting is unclear.

This study aimed to examine the accuracy of the prehospitally implemented NEWS in predicting 1-day and 30-day mortalities in an unselected EMS population. A secondary aim was to describe the causes of death in this prehospital patient population.

## Methods

This was an observational six-month cohort study in two hospital districts, Kainuu and Länsi-Pohja, in northern Finland. Permission to perform the study was obtained from both hospital districts (12Mar2014 & 8Apr2014) and the Office of Data Protection Ombudsman (719/4225/2014). The study protocol was submitted to and approved by the local ethics committee (Northern Ostrobothnia ethics committee 15/2015).

### Study setting

In Finland, the common European emergency phone number 112 is used for all (medical, fire and rescue, police) emergencies. The national dispatch authority directs the operation of the six regional emergency medical communication centres (EMCCs) in the country. For medical calls, the emergency dispatchers evaluate the calls according to a Finnish criteria-based national standardized dispatch protocol. Finnish EMSs are administrated by the hospital districts and are usually three-tiered. The first tier consists of basic life support (BLS) ambulances and first response units (FRU), such as fire engines, police, or the Border patrols. The second tier is the advanced life support (ALS) ambulance staffed with nurses or paramedics, and the third tier is a physician manned ground or helicopter unit. The EMSs in the catchment areas annually respond to approximately 35,000 incidents.

The catchment areas are home to a total of 140,000 inhabitants, representing 2.6% of the Finnish population, with a population density of 4.7 inhabitants per square km. The areas are mostly suburban and rural settings.

### Study data

Data from all prehospital emergency calls from January 1st-June 30th 2014 were reviewed from EMS databases and run sheets. Patients less than 16 years of age were excluded, as were inter-facility transports, homecare missions, and situations where the patient was not encountered. The NEWS was calculated post hoc using a statistical programme from the first clinical variables (respiratory rate, oxygen saturation, temperature, systolic blood pressure, heart rate and level of consciousness) documented by the EMS personnel on the scene. Based on the aggregate NEWS, patients were categorized into low, medium, and high risk groups according the statement by the Royal College of Physicians. [9] (See Additional file 1) Pre-hospital data, including prehospital NEWS, were coupled to the mortality data retrieved from the Causes of Death Registry maintained by Statistic Finland [11] using the patients’ national personal identification numbers.

### Statistical analyses

Statistical analyses were performed using SPSS Statistics, version 24 (IBM Corp., Armonk, NY). Summary measurements are expressed as the mean, standard deviation, and range, unless otherwise stated. When calculating the NEWS, missing values and symbols indicating normal values (e.g., ϕ, N) were considered within normal range.

We used an EMS mission (as each mission represent one patient) as the unit in all analyses and calculations. Kaplan-Meier survival curves were drawn for the 30-day mortality. Risk ratios (RR) with 95% confidence intervals were calculated and Wald’s test was used for statistical comparison with reference level. Sensitivities, specificities, positive predictive values (PPVs), negative predictive values (NPVs), positive likelihood ratios (PLRs), and negative likelihood ratios (NLRs) were calculated for 1-day and 30-day mortalities at the cut-off risks. The selection criteria with a high sensitivity and high NPV were indicated as efficient to predict patients at risk of death.

## Results

The EMS responded to a total of 16,177 missions during the study period. In 303 cases (1.9%), the unique personal identification number was lacking and a link to follow-up data was not available. After excluding these and the other emergencies fulfilling the exclusion criteria, 12,426 missions representing 7620 individual patients were included in the final analysis (see Additional file 2). Of these patients, EMS encountered 5419 patients once during the study period, while 2201 patients had two or more EMS contacts. The patients’ mean age was 63.1 (SD 22.6) years and 49.6% were female (Table 1).

**Table 1:** Demographics of all missions and deaths within 30 days of an EMS encounter categorized according to time of death

	Total	Alive Days 1–30	Deaths Day 1	Deaths Days 2–30	*P*-value
Missions, n	12.426	11.865	191	370	
Mean age, years (SD)	65.4 (20.0)	64.9 (20.1)	70.8 (15.6)	78.2 (13.1)	*< 0.001*
Male, n (%)	6283 (50.6)	6008 (50.6)	98 (51.3)	177 (47.8)	*0.55*
The time of EMS mission					*< 0.001*
00:00–07:59, n (%)	2797 (22,5)	2701 (22.8)	39 (20.4)	59 (15.9)	
08:00–15:59, n (%)	5156 (41.6)	4873 (41.1)	104 (54.5)	190 (51.4)	
16:00–23:59, n (%)	4456 (35.9)	4291 (36.2)	48 (25.1)	121 (32.7)	
Mean EMS response time from call to arrival at the scene, minutes (SD)	13.5 (10.6)	13.5 (10.6)	10.4 (8.0)	13.1 (10.6)	*< 0.001; 0.795*
Mean EMS mission time from call to arrival at the receiving facility, minutes (SD)	57.2 (31.8)	56.9 (31.7)	66.0 (33.9)	64.9 (34.2)	*0.017; < 0.001*

The records of all patient charts showed symbols indicating normal values or missing data rather than exact numeric measurements for heart rate in 7.0% of the cases, systolic blood pressure in 10.6%, oxygen saturation in 10.9%, level of consciousness in 12.7%, temperature in 23.0%, and respiratory rate in 57.4% (in the low, medium and high risk NEWS groups for respiratory rate in 63.8, 43.3 and 9.9%, respectively) of the cases**.**

In 561 cases, the patient died within 30 days from the EMS contact, representing a 4.5% overall 30-day mortality. Of these 561 cases, 191 deaths occurred within 24 h: 118 during the prehospital phase, and 73 in the hospital.

**Fig. 1:**
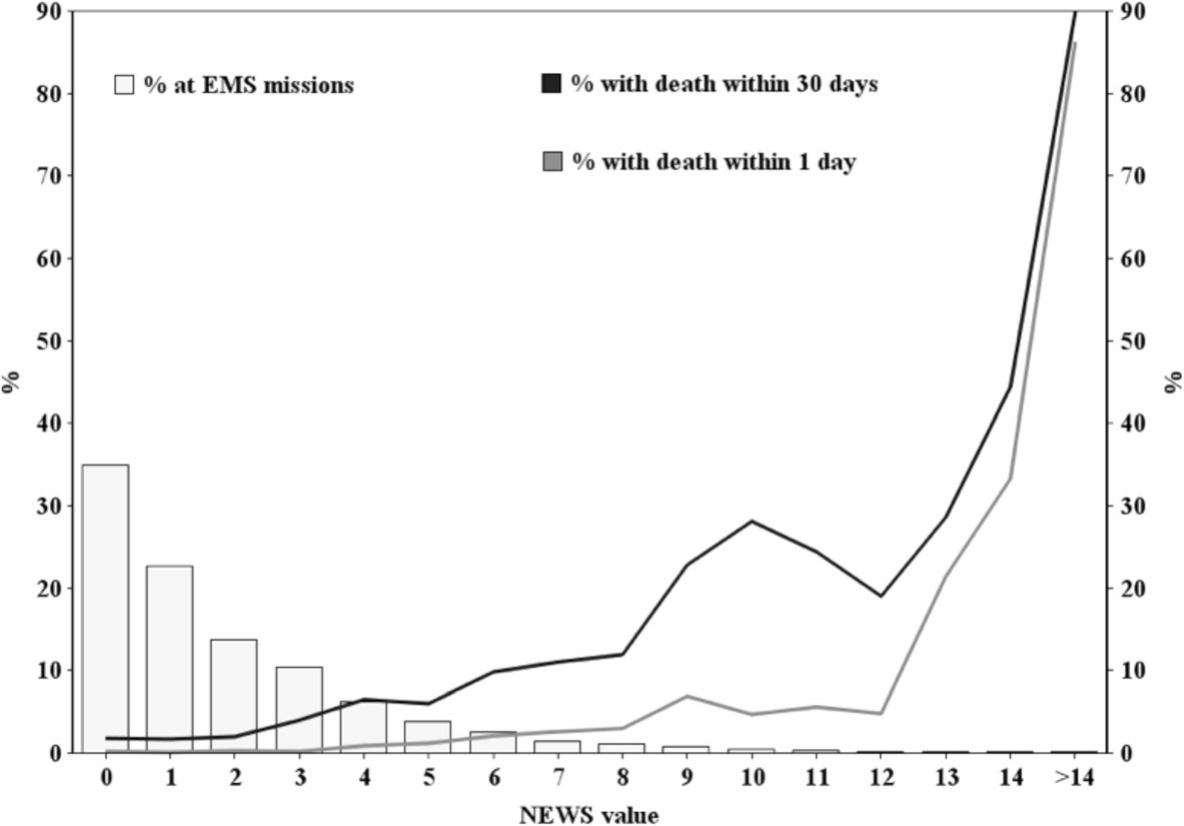
The distribution of prehospital NEWS values and the relation with 1-day and 30-day mortality

The calculated prehospital NEWS ranged from 0 to 18, with a median score of 2. In 34.9% (4342) of the cases the NEWS was 0. The distribution of the prehospital NEWS and relation with 1-day and 30-day mortalities are shown in Fig. 1, which points out that the 1-day mortality rate for NEWS values ≤12 was lower than 7% and for values ≥13 higher than 20%. 30-day mortality rate was higher than 10% with prehospital NEWS value above 6 (Fig. 1).

**Fig. 2:**
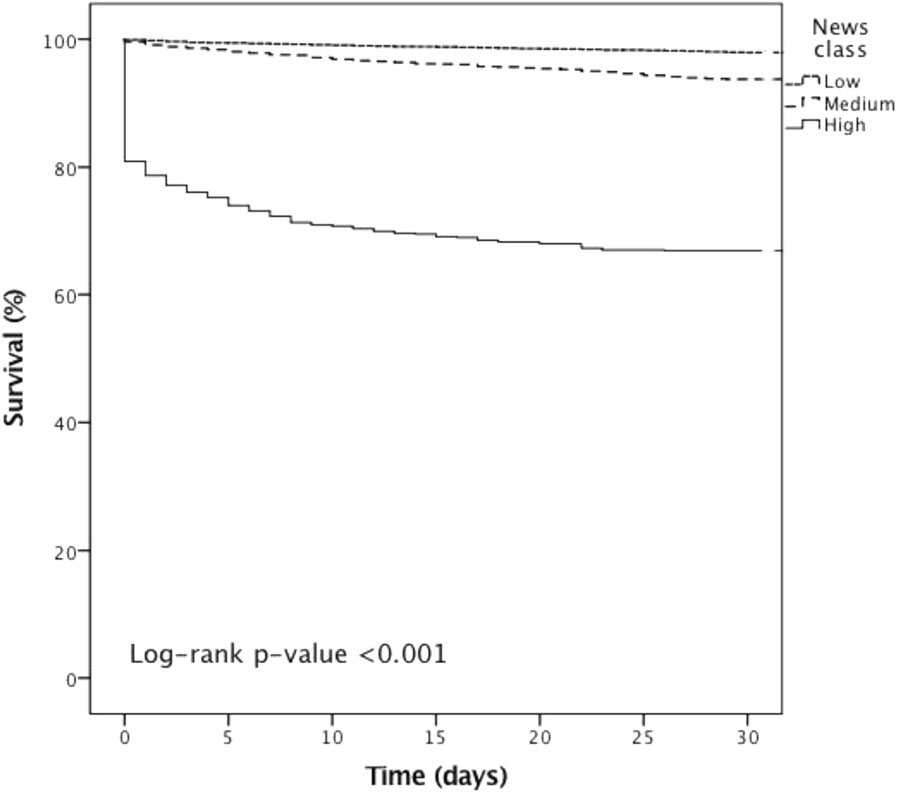
Kaplan-Meier cumulative 30-day survival curves for dispatch priorities and NEWS

The 1-day and 30-day mortalities according to prehospital NEWS are explained in more detail in Tables 2 and 3. The cumulative 30-day Kaplan-Meier survival curves are presented in Fig. 2. Patients in the high risk NEWS group demonstrated a relative risk for 1-day and 30-day mortality of 101.5 and 16, respectively, compared with the low risk group. There was a 4.4 increase in the relative risk for 1-day mortality for the medium risk NEWS group compared with the low risk category (Table 2). The 30-day mortality rate in the high risk NEWS group differed substantially from the medium and low risk groups (Fig. 2). The highest NEWS category showed a good sensitivity for 1-day mortality (0.801) but 30-day sensitivity was low (0.424). Specificities and NPVs were high. (Table 3).

**Table 2:** Mortality (1-day and 30-day) at high, medium and low risk classes categorized according to the prehospital National Early Warning Score (NEWS)

		NEWS class		
		High	Medium	Low
	Missions, n	718	1832	9876
1-day mortality	Number of deaths	153	17	21
	Mortality, % (95%CI)	21.3 (18.3 to 24.5)	0.9 (0.5 to 1.5)	0.2 (0.1 to 0.3)
	RR (95%CI)	101.5 (69.0 to 144.1)	4.4 (2.3 to 8.2)	1.0 (ref.)
	p	< 0.001	< 0.001	
30-day mortality	Number of deaths	238	115	208
	Mortality, % (95%CI)	33.1 (29.6 to 36.8)	6.3 (5.2 to 7.4)	2.1 (1.8 to 2.4)
	RR (95%CI)	16.0 (13.6 to 18.0)	3.0 (2.4 to 3.7)	1.0 (ref.)
	p	< 0.001	< 0.001	

*RR* relative risk, *95% CI* 95% confidence interval

There were 191 early 1-day deaths and 561 2–30 day deaths

Wald’s test was used for statistical comparison with reference level

**Table 3:** Sensitivity and specificity for 1-day and 30-day mortality at the cut-off high and medium risks categorized according to the prehospital National Early Warning Score (NEWS)

		NEWS class	
		High	Medium
	Missions, n	718	2550
1-day mortality	Sensitivity (95% CI)	0.801 (0.737–0.855)	0.890 (0.837–0.931)
	Specificity (95% CI)	0.954 (0.950–0.958)	0.806 (0.798–0.813)
	PPV	0.213	0.067
	NPV	0.997	0.998
	PLR	17.36	4.58
	NLR	0.21	0.14
30-day mortality	Sensitivity (95% CI)	0.424 (0.383–0.466)	0.630 (0.588–0.670)
	Specificity (95% CI)	0.960 (0.956–0.963)	0.815 (0.808–0.822)
	PPV	0.332	0.138
	NPV	0.972	0.979
	PLR	10.49	3.40
	NLR	0.60	0.46

*RR* relative risk, *95% CI* 95% confidence interval, *PPV* positive predictive value, *NPV* negative predictive value, *PLR* positive likelihood ratio, *NLR* negative likelihood ratio

Circulatory system diseases were the leading causes of death in this unselected EMS population, representing 44.2% of deaths during the 30 days after the EMS encounter. Other causes were neoplasms (16.6%), diseases of the nervous system (10.5%), and external causes (e.g. injuries, poisonings) (9.8%). The proportion of circulatory system diseases and external causes dominated in deaths within 24 h from the EMS encounter; while neoplasms and diseases of the nervous system increased during days 2–30 (Table 4).

**Table 4:** The cause of death within 1 day and 30 days of an EMS encounter, sorted by ICD-10 chapters

Chapters of ICD-10	Number of deaths	Number of deaths Day 1 (%)	Number of deaths Days 2–30 (%)
I Certain infectious and parasitic diseases	4 (0.7)	1 (0.5)	3 (0.8)
II Neoplasms	93 (16.6)	12 (6.3)	81 (21.9)
III Diseases of the blood and blood-forming organs and certain disorders involving the immune mechanism	3 (0.5)	2 (1.0)	1 (0.3)
IV Endocrine, nutritional and metabolic diseases	5 (0.9)	3 (1.6)	2 (0.5)
V Mental and behavioural disorders	17 (3.0)	5 (2.6)	12 (3.2)
VI Diseases of the nervous system	59 (10.5)	13 (6.8)	46 (12.4)
IX Diseases of the circulatory system	248 (44.2)	110 (57.6)	138 (37.3)
X Diseases of the respiratory system	31 (5.5)	3 (1.6)	28 (7.6)
XI Diseases of the digestive system	38 (6.8)	9 (4.7)	29 (7.8)
XIII Diseases of the musculoskeletal system and connective tissue	3 (0.5)	0 (0.0)	3 (0.8)
XIV Diseases of the genitourinary system	4 (0.7)	1 (0.5)	3 (0.8)
XVIII Symptoms, signs and abnormal clinical and laboratory findings, not elsewhere classified	1 (0.2)	1 (0.5)	0 (0.0)
XX External causes of morbidity and mortality	55 (9.8)	31 (16.2)	25 (6.5)
Total	561 (100.0)	191 (100.0)	370 (100.0)

## Discussion

This study showed a rather twofold accuracy of using NEWS in the prehospital patient population to predict short-term mortality. The high risk NEWS category could predict 8 in 10 early deaths within 24 h, but failed to acceptably predict 30-day mortality due to a high rate of false negatives. Since the prehospital NEWS is often based on the single values measured in the brief time interval, it may not adequately discriminate patients who may deteriorate later. Although NEWS is designed and used to detect patients who may develop critical illness, and not directly intended to predict mortality, it is of interest to discuss the use of NEWS to support prehospital decision-making.

Overall, prehospital NEWS showed low sensitivity for 30-day mortality. This may be explained by the patients’ higher ages and the greater proportion of deaths occurring 2–30 days after EMS contact, caused by chronic diseases (e.g. neoplasms and diseases of the nervous system). A Scottish study, describing a cohort of 1684 transported EMS patients, identified high-risk prehospital NEWS patients with 48-h and 30-day sensitivities for mortality of 0.71 and 0.40, [10] respectively, which resembles our results. However, compared with our study, only in-hospital deaths were included, and follow-up after discharge was incomplete, causing a potential underestimation of the mortality rate.

Our present results showed that the overall 1-day mortality in the EMS population was 1.5%, whereas 30-day mortality was 4.3%. This concurs with a recent population-based Danish study, which reported 1.8 and 4.7% for the 1-day and 30-day mortalities, respectively, of EMS patients transported to the hospital. [12] A Swiss population-based study demonstrated a 48-h mortality rate of EMS patients as high as 11%. [13] However, those figures are not fully comparable to our result due to differences between countries in the dispatch protocols and the EMS systems.

Interestingly, our results differ from the in-hospital cohort related to 1-day mortality. [14] The study by Smith et al. showed that 1-day mortality for hospital patients increased promptly as the NEWS value exceed 7, [14] while in our material the increase in 1-day mortality occurred with NEWS value greater than 12. However, in our series the 30-day mortality was above 10% in those patients with NEWS value higher than 6.

The diagnostic pattern of causes of death were different between 1-day and 2–30 –day deaths. In both groups, the deaths caused by the cardiovascular diseases dominate, but the proportion of traumatic deaths was greater within 1-day. Although prehospital emergency care has developed significantly over the last decades, a notable proportion of traumatic deaths are still estimated to be preventable. [15] A large number of deaths due to neoplasms and diseases of the nervous system indicate that many chronically ill patients need EMS during their last days of life, either due to acute exacerbation or to deterioration of general condition.

A somewhat surprising finding was that in more than half of all patient charts, a numeric number for respiratory rate was missing although it was recorded as normal. Respiratory rate is an important parameter in several scoring systems, and it may be that the significance of accurate documentation has received too little attention during training. The importance of documenting this has been recognised in the development of the national electronic EMS charting system where respiratory rate will be a compulsory data field to fill.

### Limitations and strengths

An evident limitation of this study was that in 303 cases, a Finnish personal identification number was missing and, therefore, mortality data could not be retrieved. These patients may have had a higher or lower mortality, and thus caused a bias in the study. However, they represent only 1.9% of the EMS calls. In addition, when calculating the NEWS, missing values and markings indicating normal values were considered normal; thus, some patients might have been incorrectly classified with a lower risk. Especially in respiratory rate, paramedics tend to mark value as normal if the breathing is considered normal according to the clinical judgement. However, in daily practice, patients with more severe disease are more comprehensively assessed; therefore, excluding a significant proportion of low risk patients with incomplete data would distort the results. In addition, in this study, there were a number of patients with several EMS contacts within a short period of time, which may have distorted the results. Finally, the results may not apply to systems with different population densities or morbidity indexes.

The main strength of this study was that we included an entire prehospital patient population in two hospital districts. The EMS entity is better described, if not only focused on specific conditions. Both the EMS databases as well as the Death Cause Registry were complete with no missing information. The quality of the Finnish Death Cause Registry is also high [16].

### Clinical significance

In Finland, the prehospital NEWS is designed to be implemented in the national EMS database. Our results imply that NEWS in the prehospital setting may be of value when assessing the mortality risk within 24 h and hence immediate need for medical care. However, based on this cohort, NEWS alone cannot guide decision-making about the urgency of transport, the destination of transport, or whether to transport or not, and needed to be further studied. Other tools are needed to predict long-time mortality risk and may include other risk scoring systems applied at the emergency units.

## Conclusion

In prehospital environment, the high risk NEWS category was associated with 1-day mortality well above that of the medium and low risk NEWS categories. This effect was not as noticeable for 30-day mortality. The prehospital NEWS may be useful tool for recognising patients at early risk of death, allowing earlier interventions and responds to these patients.

## Additional files

Additional file 1: NEWS usage; calculation of NEW-score and definition of the clinical risk. (DOCX 137 kb)

Additional file 2: Flow chart of study cohort. (PDF 35 kb)

## Abbreviations

ALS: Advanced life support
BLS: Basic life support
EMCC: Emergency medical communication centre
EMS: Emergency medical service
FRU: First responding unit
NEWS: National Early Warning Score
NPV: negative predictive value
PPV: positive predictive value

## References

[1] Ornato JP (2009). Science of emergency medical dispatch. Circulation.

[2] Goldhill D, R., McNarry A, F. Physiological abnormalities in early warning scores are related to mortality in adult inpatientsâ€. BJA (2004). Br J Anaesth.

[3] Wilson S, Cooke M, Morrell R, Bridge P (2002). Allan T. A systematic review of the evidence supporting the use of priority dispatch of emergency ambulances. Prehosp Emerg Care..

[4] Williams TA, Tohira H, Finn J, Perkins GD, Ho KM (2016). The ability of early warning scores (EWS) to detect critical illness in the prehospital setting: a systematic review. Resuscitation.

[5] Schoos MM, Sejersten M, Baber U, Treschow PM, Madsen M, Hvelplund A (2015). Outcomes of patients calling emergency medical Services for Suspected Acute Cardiovascular Disease. Am J Cardiol.

[6] Sasson C, Rogers MAM, Dahl J, Kellermann AL (2010). Predictors of survival from out-of-hospital cardiac arrest. Circ Cardiovasc Qual Outcomes.

[7] Rawshani A, Larsson A, Gelang C, Lindqvist J, Gellerstedt M, Bång A (2014). Characteristics and outcome among patients who dial for the EMS due to chest pain. Int J Cardiol.

[8] Brice JH, Garrison HG, Evans AT (2000). Study design and outcomes in out-of-hospital emergency medicine research: a ten-year analysis. Prehosp Emerg Care.

[9] Royal College of Physicians (2012). National Early Warning Score (NEWS): standardizing the assessment of acute-illness severity in the NHS. Report of a working party. London.

[10] Silcock DJ, Corfield AR, Gowens PA, Rooney KD (2015). Validation of the National Early Warning Score in the prehospital setting. Resuscitation.

[11] Finland S (2015). Statistical database..

[12] Christensen EF, Larsen TM, Jensen FB, Bendtsen MD, Hansen PA, Johnsen SP (2016). Diagnosis and mortality in prehospital emergency patients transported to hospital: a population-based and registry-based cohort study. BMJ Open.

[13] Pittet V, Burnand B, Yersin B, Carron P (2014). Trends of pre-hospital emergency medical services activity over 10 years: a population-based registry analysis. BMC Health Serv Res.

[14] Smith GB, Prytherch DR, Meredith P, Schmidt PE, Featherstone PI (2013). The ability of the National Early Warning Score (NEWS) to discriminate patients at risk of early cardiac arrest, unanticipated intensive care unit admission, and death. Resuscitation.

[15] Bakke HK, Wisborg T (2017). The trauma chain of survival — each link is equally important (but some links are more equal than others). Injury.

[16] Sund R (2012). Quality of the Finnish hospital discharge register: a systematic review. Scand J Public Health.

