# Supplementary figures and images for: Does the prehospital National Early Warning Score predict the short-term mortality of unselected emergency patients?
Source: Scand J Trauma Resusc Emerg Med. 2018 Jun 7;26:48. doi: 10.1186/s13049-018-0514-1 (PMC5992854; doi:10.1186/s13049-018-0514-1)

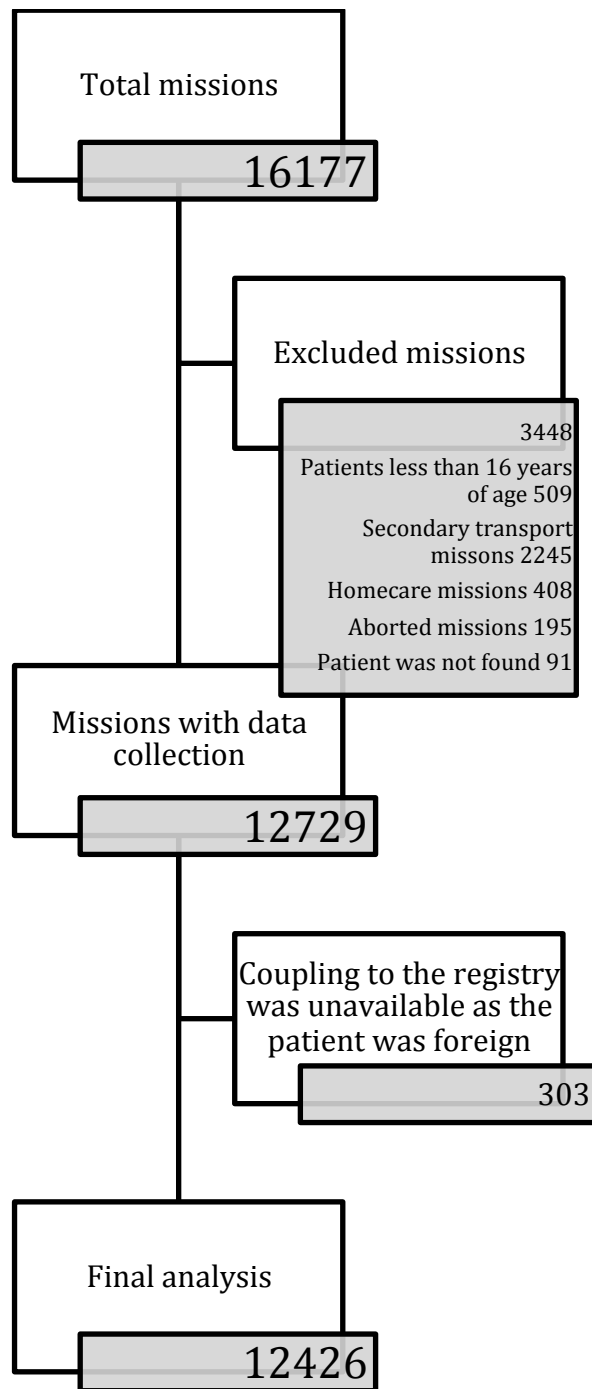

Supplement: Supplementary file 2 — Flow chart of study cohort. (PDF 35 kb) [file 13049_2018_514_MOESM2_ESM.pdf]
